# Issues with the European Pharmacopoeia Quality Control Method for ^99m^Tc-Labelled Macroaggregated Albumin

**DOI:** 10.3390/molecules27133997

**Published:** 2022-06-22

**Authors:** Svend Borup Jensen, Lotte Studsgaard Meyer, Nikolaj Schandorph Nielsen, Søren Steen Nielsen

**Affiliations:** 1 Department of Nuclear Medicine, Aalborg University Hospital, 9100 Aalborg, Denmark; lsn@rn.dk (L.S.M.); nsn@rn.dk (N.S.N.); ssn@rn.dk (S.S.N.); 2 Department of Chemistry and Biochemistry, Aalborg University, 9220 Aalborg, Denmark

**Keywords:** technetium-99m labelled macroaggregated albumin [^99m^Tc]Tc-MAA, European Pharmacopoeia, lung perfusion scintigraphy, quality control, EANM recommendation

## Abstract

Technetium-99m macroaggregated albumin ([^99m^Tc]Tc-MAA) is an injectable radiopharmaceutical used in nuclear medicine for lung perfusion scintigraphy. After changing to a new batch of macroaggregated albumin (MAA), we saw unwanted uptake in the liver and spleen. The batch was therefore tested by both the supplier and us and we found it to comply with the requirements of the European Pharmacopoeia (Ph. Eur.). However, a simple comparison between the problematic batch and a batch supplied by another manufacturer showed that there was a significant difference. The quality testing showed a higher number of small particles in the problem encumbered MAA batch with unwanted in vivo uptake. In this article we present a simple method of testing for particle size of [^99m^Tc]Tc-MAA, which gives a good indication of how the radioactive drug performs in vivo. We argue that the quality control method described in the Ph. Eur. should be changed. The changes will improve concordance between the laboratory analyzes and what is seen in vivo in human lung perfusion scintigraphy. Furthermore, we hope that the MAA suppliers without delay will replace their release procedure to be in accordance with the method described in this article.

## 1. Introduction

Technetium-99m labelled macroaggregated albumin ([^99m^Tc]Tc-MAA) is, at our hospital, used to perform lung perfusion scintigraphy on suspicion of pulmonary embolism and prior to operation for lung cancer to be able to calculate postoperative lung function. Our customary supplier of MAA labelling kits notified us that they could not supply us with labeling kits for preparation of [^99m^Tc]Tc-MAA, due to production issues.

We applied and were granted a compassionate user permission for MAA from Medi-Radiopharma, Hungary, from the Danish Medical Agency. The first batch was received and performed as expected. However, the second batch of the MAA labelling kits, received in July 2020, resulted in unwanted radioactivity in the liver and spleen in many patients.

Additional information on this second MAA batch: the temperature data logger that accompanied the parcel revealed that the batch had been too warm during the shipment to Denmark. The maximum temperature should not have exceeded 8 °C but had been 10 °C for 12 h. We consulted Medi-Radiopharma who replied that based on their stability data for MAA, they could conclude that the temperature exceedance did not affect the product quality. Our standard TLC quality control of the [^99m^Tc]Tc-MAA batch revealed nothing abnormal, and it complied with specification. The [^99m^Tc]Tc-MAA was therefore released for clinical use. Shortly after, our doctors detected unusual uptake in the liver and spleen and started questioning the quality of the product.

The TLC analysis used for the release of the batch was repeated and again the batch complied with our release specification. The common TLC release analysis only differentiates between [^99m^Tc]Tc-MAA and free [^99m^Tc]pertechnetate. However, looking at the in vivo uptake pattern, it seemed likely that it could be an unusually higher number of small particles, which had been labeled and then caused the abnormal activity pattern in the perfusion scintigraphy. Generally, we rely on the company analysis for the particle size analysis. The release document stated that 90.34% of the MAA particles were between 10–100 µm and that no particles were above 150 µm, measured by optical microscope as described in the monograph on Tecchnetium (^99m^Tc) macrosalb injection of the European Pharmacopoeia (Ph. Eur.).

The monograph says that 90% of the particles typically should have diameters between 10–100 µm [1]. However, Ph. Eur. does not describe an exact method by which one can measure this. Ph. Eur suggests that one can use an optical microscope to verify whether the particles are not too big, but not that one can use an optical microscope to determine if the particles are too small, as Medi-Radiopharma did. Ph. Eur. suggests an indirect test method for particles size, it is a filter method (pore size of 3 µm). This method should reveal if there are too many small radiolabelled particles. The requirements of the United States Pharmacopeia on ^99m^Tc-labeled macroaggregated albumin (MAA) are very similar to those of the European Pharmacopeia it states that 90% of the particle size should have a diameter between 10 and 90 μm and none of the observed particles have a diameter greater than 150 μm [2]. This range was chosen to ensure that particles smaller than the 7–8 μm diameter of capillaries would be absent, eliminating the possibility of localization in the brain, kidneys, and other internal organs. The limit value at the high end, was chosen to minimize the risk of occlusion of larger vessels, especially in the patient with pulmonary artery hypertension, in which case vasoconstriction is present [3].

MAA is prepared from human serum albumin under carefully controlled conditions of pH, time, temperature, agitation, and reagent concentration to insure correct particle size formation [3].

Reviewing the literature on macroaggregated albumin particle sizes revealed a couple of studies which examined the particle size distributions in commercially available MAA brands [4,5]. And an older study which compared the pharmaceutical quality of a laboratory made MAA with commercially available products [6]. All three studies applied microscope methods for particle size determination. The conclusion was that the mean particle size was similar for all the five macroaggregated albumin preparations tested, but the actual particle size distribution varied considerably among the products tested [4,5]. In the study from Hung and coworkers [5], they also examined and found particle sizes intervariance among the best-performing kit and they concluded that the particle sizes may affect the accuracy and the reproducibility of their patient studies.

We decided to examine the [^99m^Tc]Tc-MAA by filtration, not just with one size filter as described in Ph. Eur., but with four different pore size filters. We found the problem encumbered [^99m^Tc]Tc-MAA, called [^99m^Tc]Tc-Makro-Albumon, to comply with the Ph. Eur. However, comparing the particle sizes between this batch and a different batch supplied by a different vendor of MAA, it was noticeable that the distribution of particle size was significantly different.

On the basis of the in vivo human data and of the results of our particle size experiments, we decided to discard the problem encumbered batch. We have informed the Danish Medicines Agency that we believe that the quality control described in the Ph. Eur. should be changed, so that it will identify batches which will result in unwanted uptake in the liver and spleen. The Danish Medicines Agency has agreed to look at our data (private mail communication between us and the Danish Medicines Agency), but since it may take some time to change the Ph. Eur., we hope that the MAA producers will comply with the proposed changes for the benefit of the patients.

## 2. Results

### 2.1. Labeling

Radioactive labeling with [^99m^Tc]pertechnetate is a one pot chelation reaction with less than 2% unreacted [^99m^Tc]pertechnetate detected after chelation. For comparison two MAA precursors were labeled, both the problem encumbered batch from Medi-Radiopharma and a batch from CIS Bio International France, called Pulmocis The radiolabeled [^99m^Tc]Tc-MAA preparations are designated as respectively [^99m^Tc]Tc-Makro-Albumon and [^99m^Tc]Tc-Pulmocis.

### 2.2. Particle Size Determined by a Microscope

Determination of particle size by a microscope was performed by Medi-Radiopharma Hungary and revealed that an average of 92.8% of the particles have a size between 10–100 µm (10 vials examined), see Table 1.

### 2.3. Indirect Particle Size Determination

An indirect determination of particle size by a filtration method applying four different pore size filters (pore size of 3, 5, 8 and 10 µm) was performed. The test results from three examinations of the problem encumbered [99mTc]Tc-Makro-Albumon preparation is given in Table 2. The test results from three examinations of the [99mTc]Tc-MAA preparations from Pulmocis labelling kits is given in Table 3.

## 3. Discussion

**Figure 1:**
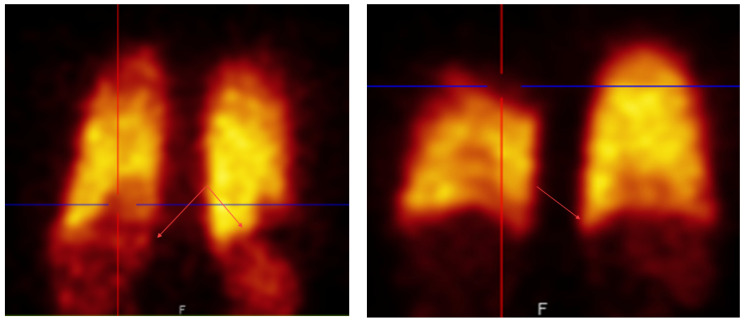
Two examples of abnormal hepatic and spleen uptake seen in lung perfusion scintigraphy when using the problematic [^99m^Tc]Tc-MAA batch, called [^99m^Tc]Tc-Makro-Albumon.

This study started because our doctors noticed abnormal uptake in the lung perfusion scintigraphy when using a new batch of [^99m^Tc]Tc-MAA, Figure 1.

To evaluate this observation, an experienced nuclear medicine specialist randomly selected 55 [^99m^Tc]Tc-MAA perfusion scintigrams made after injection of [^99m^Tc]Tc-Makro-Albumon perfusion reviewed them. None of the patients had known history of cardiac shunt or liver cirrhosis which can be physiological causes of right-to-left shunting causing trapping of particles in organs like the liver and spleen. In 78% of the scintigraphies, he identified liver or spleen uptake which is not normally seen using [^99m^Tc]Tc-MAA prepared from labelling kits supplied by a different vendor without the higher number of small particles. In fact, three of the 55 reviewed [^99m^Tc]Tc-Makro-Albumon scanned patients had an earlier scintigram with a [^99m^Tc]Tc-MAA prepared using a labeling kit supplied from a different vendor, and none of these earlier scintigrams showed unwanted activity in the liver or spleen.

The patients with abnormal uptake (78%) were divided into 3 groups: visible, moderate and clearly increased uptakes. In 33% of all the scintigrams, the uptake was classified as a moderate uptake so in one out of three patients the unwanted uptake slightly affected the study interpretation. Furthermore, a clearly increased uptake in the liver or spleen was found in four out of the 55 of the patients (7%) and this uptake affected the interpretation of the study.

We could not explain the unwanted uptake using our commonly applied quality control method, which is a radio-TLC. The radio-TLC revealed that less than 2% was free [^99m^Tc]pertechnetate, which is in line with what we usually see. These results indicaed that more than 98% of the radioactivity could be in the form of [^99m^Tc]Tc-MAA.

Our attention, therefore, turned to the particle size of the problem encountered MAA batch. Too many small particles could probably explain the unwanted lung and spleen uptake [7]. Was the [^99m^Tc]Tc-Makro-Albumon from Medi-Radiopharma, Hungary within specification? Our suspicion was increased by the fact that the kit, during shipment, exceeded the threshold temperature value and also by the fact that the certificate of analysis stated that only 90.3% of the particles was between 10–100 µm. According to the certificate of analysis 90% or more of the particles must be between 10–100 µm, so the batch was only just within specification.

Due to the unwanted uptake in the liver and spleen in human scans, we started a dialog with the supplier, who asked us to return the problematic batch (no. MA-200405-1) for reanalysis. We returned 12 kits. They tested ten kits of the troublesome batch. On the original certificate of analysis for MA-200405-1, they stated that 90.3% of the particles were within 10–100 µm. For results of the reanalysis, see Table 2. The figures were between 91.2% and 94.9% (mean 92.8%), so all the ten vials reexamined performed better than the original vial which were used for batch release. The Ph. Eur. also states that on a total of 5000 particles examined no more than ten particles must have a dimension bigger than 100 µm and that no particles must have a dimension above 150 µm. Medi-Radioharma found that batch no. MA-200405-1 complies with Ph. Eur.’s specifications on all accounts.

We decided to perform our own particle size test. The Ph. Eur. states that 90% of the radioactivity must be retained on a polycarbonate membrane filter, pore size 3 µm [1]. We hypothesized that we could gain more information on the particle size of the denatured albumin particles in the MAA labelling kits if we used several filters with different pore sizes. We, therefore, decided to apply four different pore size filters (3, 5, 8, and 10 µm) in the experiment. All the filters had a diameter of 25 mm.

For comparison, we tested two different [^99m^Tc]Tc-MAA batches, both the problematic batch from Medi-Radiopharma here called [^99m^Tc]Tc-Makro-Albumon and a batch from CIS Bio International France here called [^99m^Tc]Tc-Pulmocis.

**Figure 2:**
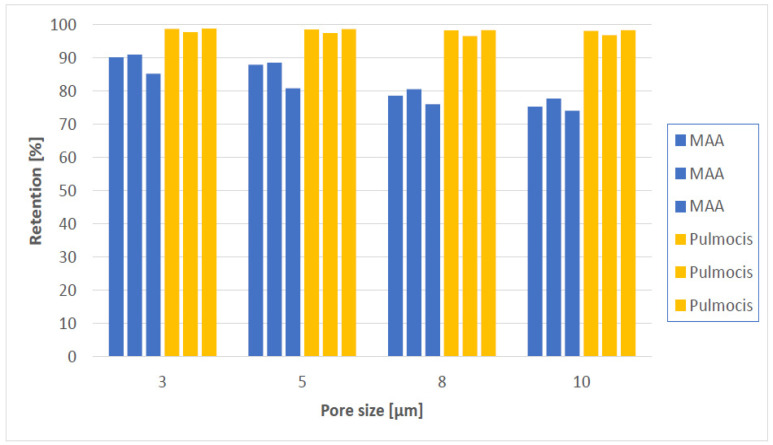
Filter test results from applying the four pore size filters on three [^99m^Tc]Tc-Makro-Albumon (MAA) and three [^99m^Tc]Tc-Pulmocis (Pulmocis) products.

If we look at [^99m^Tc]Tc-Makro-Albumon (named MAA in Figure 2) first, 90.1% of the radioactivity is retained on polycarbonate membrane filter with a pore size 3 µm in the first experiment and 90.9% in the second experiment. That is in both cases within the acceptance limit according to the Ph. Eur.

In the last experiment, only 85.2% of the radioactivity was retained on a 3 µm pore size filter. That was below the required 90%. But the Macro-Albumon kits had exceeded their expiration date by about a month when we did experiment 3, so the kit would not have been used in humans anyway.

In other words, the [^99m^Tc]Tc-Makro-Albumon batch still complied with the requirements set out in the Ph. Eur [1]. We therefore cannot claim compensation because the [^99m^Tc]Tc-Makro-Albumon batch meets the requirements described by the Ph. Eur., even though we do not think the batch should be used in humans.

The recommendation described in the European Association of Nuclear Medicine, (EANM) guidelines for ventilation/perfusion scintigraphy from 2009 [8] or 2019 [9] both say that the size of the aggregates of a [^99m^Tc]Tc-MAA preparation should be within 15–100 µm not within 10–100 µm as the Ph. Eur. suggests. We find it peculiar that the Ph. Eur. and EANM do not recommend the same interval for particle size.

An examination of our human data, clearly showed that particle size is important in connection with the MAA particles to lodge in the pulmonary capillaries and in the precapillary arterioles. The question is, would the [^99m^Tc]Tc-Makro-Albumon batch with the unwanted liver and spleen uptake have failed the quality control if it was required that that 90% of the particles had to be in-between 15–100 µm instead of 10–100 µm? We cannot say for sure, but it is likely since the batch was close to failing. The certificate of analysis says that only 90.3% of the particles were between 10–100 µm. If we look at the filtration analysis of the [^99m^Tc]Tc-Makro-Albumon for the two experiments where the batch still meets the expiration date (experiment 1 and 2, Table 2, it was found that approximately 90.5% of the radioactivity was retained applying a 3 µm pore size filter, 88.2% for a 5 µm pore size filter, 79.5% for a 8 µm pore size filter and 76.5% for a 10 µm pore size filter. Thus, 90.5% − 88.2% = 2.3% of the particles were retained on a 3 µm pore size but not on a 5 µm pores, 11% were retained by 3 µm but not by 8 µm pore, and 14% were retained by 3 µm but not by 10 µm pores. Regardless of which pore size filters were used more than 90% of the [^99m^Tc]Tc-Pulmocis particles were retained.

Looking at the four filters and the six experiments in Figure 2, the two products have a measurable difference in particle size composition. In this article we present a simple analytical method which can distinguish between a [^99m^Tc]Tc-MAA batch that results in unwanted lung and spleen uptake and a batch that does not. The method is so simple that the test can be performed at any nuclear medicine department and does not require expensive equipment. To do the test, one just needs a dose calibrator and a polycarbonate membrane filter. Instead of using a polycarbonate membrane filter with a pore size of 3 µm, one could do the test with a filter having a slightly bigger pore size. In our case one could use either a 5, 8 or 10 µm with the same results. The batch with the unwanted liver and spleen uptake would have failed, and one could avoid using a [^99m^Tc]Tc-MAA batch with unwanted uptake in the lung and spleen in humans.

## 4. Materials and Methods

### 4.1. Materials

Macro-Albumon labelling kits were purchased from Medi-Radiopharma, Érd, Hungary through Wiik Pharma, Hinnerup, Denmark. Pulmocis labelling kits were purchased from CIS Bio International, Saclay, France via Dupharma, Kastrup, Denmark. The technetium-99m generator was an Ultra-TechneKow FM Generator from Curium, Petten, Netherland bought through GE, Brøndby, Denmark. Acetone (GC quality, purity ≥ 99.5%) was purchased from Sigma-Aldrich Søborg, Denmark. Silica gel 60 Aluminum TLC plates and the four different types of Merck Isopre Polycarbonate membrane filters, all 25 mm in diameter, were purchased from Merck Life Science A/S, Søborg, Denmark with pore size diameters of respectively 3.0 µm, 5.0 µm, 8.0 µm and 10.0 µm (Merck IsopreTM Membrane Filter, 3.0 µm, Item number: TSTP02500/Lot R9MA87321), (Merck IsopreTM Membrane Filter, 5.0µm Item number.: TMTPO2500/Lot.ROEB65842), (Merck IsopreTM Membrane Filter, 8.0µm Item number. TETPO2500/LotROMB21842), (Merck IsopreTM Membrane Filter, 10.0µm Item number. TOTPO2500/LotROHB80056). Radio-TLC was performed using a Scan-RAM radio-TLC scanner from Lablogic, Sheffield, UK. Microscopic analysis of the particle size was done by Medi-Pharma, Érd, Hungary. For measurement of radioactivity a gamma counter (Wizard 2/2480, Perkin Elmer, Skovlunde, Denmark) and a Capintec (CRC^®^-55tR), dose calibrator (Hoy Scientific, Hadsund, Denmark) were used.

### 4.2. The Preparation of [^99m^Tc] Macroaggregated Albumin ([^99m^Tc]Tc-MAA)

The production of [^99m^Tc]Tc-Makro-Albumon and [^99m^Tc]Tc-Pulmocis followed the manufacturer’s package inserts.

The ^99^Mo/^99m^Tc generator had been eluted within 24 h before it was used for preparation of [^99m^Tc]Tc-Makro-Albumon or [^99m^Tc]Tc-Pulmocis. The generator eluate had been checked for molybdenum-99 breakthrough prior to labeling. The ^99^Mo/^99m^Tc generator was eluted, the radioactivity was measured in a dose calibrator and an appropriate part of the eluate was then used for labeling shortly after (see below).

#### 4.2.1. [^99m^Tc]Tc-Makro-Albumon

[^99m^Tc]Tc-Makro-Albumon was prepared with 3700 MBq Tc-99m in 8 mL saline solution (9 g/L NaCl) without a breather needle. After adding the [^99m^Tc]pertechnetate solution to the macroaggregated albumin labeling vial, an equivalent volume of nitrogen was withdrawn to avoid excess pressure in the vial. The kit was turned upside down after the radioactivity had been added and placed on a tilting device for two minutes. Then the kit was left standing at room temperature for 20 min before it was ready for use.

#### 4.2.2. [^99m^Tc]Tc-Pulmocis

[^99m^Tc]Tc-Pulmocis was prepared with 3700 MBq Tc-99m in a 10 mL saline solution (9g/L NaCl) without a breather needle. After adding the [^99m^Tc]pertechnetate solution to the macroaggregated albumin labeling vial, an equivalent volume of nitrogen was withdrawn to avoid excess pressure in the vial. The kit was then gently turned upside down for two minutes, and it was then left at room temperature for 15 min, after which it was stored refrigerated until usage.

### 4.3. Quality Control of the [^99m^Tc] Macroaggregated Albumin ([^99m^Tc]Tc-MAA)

#### 4.3.1. Microscopic Analysis

Determination of particle size by microscopic analysis was performed by Medi-Radiopharma, following Ph. Eur. [1], after dilution of the preparations to a level where the number of particles was just low enough for individual particles to be distinguished. Using a syringe fitted with a needle having a caliber not less than 0.35 mm, a suitable volume was placed in a counting chamber. The preparation was allowed to settle for one minute, a cover-slide was applied carefully without squeezing the sample. The examined area covered at least 5000 particles.

The Ph. Eur. [1] explains how to determine large molecules using a microscope. However, Medi-Radiopharma uses this test to identify both particles that are too big and those which are too small.

#### 4.3.2. Non-Filterable Radioactivity

A polycarbonate membrane filter with a 13–25 mm diameter and a pore size of 3 µm was used as prescribed by the Ph. Eur. However we decided to use four different pore size filters (3, 5, 8, and 10 µm) in the experiment. All 4 polycarbonate membrane filters had a diameter of 25 mm.

For comparison and to test the method, we tested both the [^99m^Tc]Tc-Makro-Albumon from Medi-Radiopharma and the similar radiopharmaceutical [^99m^Tc]Tc-Pulmocis from Dupharma. Both products where gently turned upside down a couple of times before taking 4 × 0.2 mL out for the four analyses. The 0.2 mL product solution was added to each of the above-described filters followed by 20 mL saline (9 g/L). The solution was passed gently through the filter. To calculate the retained fraction, both the filtered solvent and the filter were measured in a dose calibrator. The results are presented in Table 2 and Table 3.

#### 4.3.3. Radio-TLC Methods for the Quantitative Determination of [^99m^Tc]Tc-MAA

The product mixture was turned gently upside down a couple of times before transferring 5 µL of the product mixture onto a 10 cm long Silica gel-60 TLC plate one cm from the bottom. The TLC plate was placed in a beaker, the bottom of the beaker was covered with acetone (0.5 cm). When the solvent front reached about 9cm, the TLC-plate was removed from the beaker and examined by a radio TLC scanner.

The [^99m^Tc]Tc-MAA does not move, whereas free [^99m^Tc]pertechnetate does.

There were found below 2% free [^99m^Tc]pertechnetate in both the [^99m^Tc]Tc-Makro-Albumon and the [^99m^Tc]Tc-Pulmocis production.

### 4.4. In Vivo Evaluation in Patients

An experienced nuclear medicine specialist reviewed 55 [^99m^Tc]Tc-MAA perfusion scintigraphy. These studies represented consecutive examinations from 15 October 2020 until 19 November 2020. We also randomly retrieved lung-perfusion scintigraphy of the month prior to the unwanted uptake in the liver and spleen.

## 5. Conclusions

We have shown that the limit values described in Ph. Eur. are too wide and are unable to identify a [^99m^Tc]Tc-macroaggregated albumin batch with unwanted lung and spleen uptake in more than 75% of patients examined. The requirement that at least 90% of the particles must be larger in size than 10 was met for the batch with the unwanted liver and spleen uptake, as shown by the microscopic analysis of the Hungarian manufacturer. The aim must be to identify a batch like that before it gets into patients. There is therefore a need for tighten the requirement. A cautious recommendation for the guidelines could be that 95% of the particles must be larger in size than 10 µm. Moreover, we have shown that a simple and inexpensive filter analysis using polycarbonate membrane filter (pore size 5, 8 or 10 µm) in our case can distinguish between the batches performing as it should in vivo from the batch with the unwanted lung and spleen uptake.

This article will be sent to the Danish Medicines Agency with the aim of changing the European Pharmacopoeia Quality Control Method for 99mTc-labeled of macroaggregated albumin. We suggest changing the wording of the Ph. Eur. to that 95% of the particles should be between 10–100 µm, and that 95% of the particles should be retained on a polycarbonate membrane filter with a pore size of 3 µm (or alternatively that 90% must be retained on a filter with pore size 5, 8 or 10 µm).

## Figures and Tables

**Table 1:** Particle size determination by Medi-Radiopharma, Hungary on the problem cumbered [^99m^Tc]Tc-Makro-Albumon batch by a microscopic analysis of the contents of ten labelled kits.

Test	10–100 µm	100–150 µm	>150 µm
1	92.4%	0	0
2	92.5%	2	0
3	91.3%	1	0
4	93.8%	2	0
5	91.2%	2	0
6	91.8%	1	0
7	93.5%	5	0
8	92.3%	7	0
9	93.9%	8	0
10	94.9%	4	0
Mean	92.8%	3.2	0
Specification	>90%	max:10 pcs *	0 pcs *

* A minimum of 5000 particles (pcs) examined.

**Table 2:** Table showing filter test results from three examinations of the problem encumbered [^99m^Tc]Tc-Makro-Albumon preparation.

**Filter Pore Size**	**Filter (MBq)**	**Filtered Solution (MBq)**	**Filter/(Filter + Filtered Solution) (%)**
3 µm	84.3	9.26	90.1
5 µm	76.3	10.51	87.9
8 µm	67.1	18.26	78.6
10 µm	65.0	21.27	75.3
**Filter Pore Size**	**Filter (MBq)**	**Filtered Solution (MBq)**	**Filter/(Filter + Filtered Solution) (%)**
3 µm	83.8	8.39	90.9
5 µm	69.9	9.11	88.5
8 µm	56.4	13.62	80.5
10 µm	56.1	16.07	77.7
**Filter Pore Size**	**Filter (MBq)**	**Filtered Solution (MBq)**	**Filter/(Filter + Filtered Solution) (%)**
3 µm	71.2	12.29	85.2 *
5 µm	71.3	16.86	80.8 *
8 µm	66.3	20.90	76.0 *
10 µm	65.3	22.90	74.0 *

* The expiration date was exceeded by about one month.

**Table 3:** Table showing filter test results from three [^99m^Tc]Tc-MAA preparations from Pulmocis labelling kits.

**Filter Pore Size**	**Filter (MBq)**	**Filtered Solution (MBq)**	**Filter/(Filter + Filtered Solution) (%)**
3 µm	113.0	1.46	98.7
5 µm	112.2	1.76	98.5
8 µm	111.7	2.07	98.2
10 µm	111.4	2.11	98.1
**Filter Pore Size**	**Filter (MBq)**	**Filtered Solution (MBq)**	**Filter/(Filter + Filtered Solution) (%)**
3 µm	114.2	2.66	97.7
5 µm	109.5	2.97	97.4
8 µm	93.6	3.43	96.5
10 µm	91.6	3.05	96.8
**Filter Pore Size**	**Filter (MBq)**	**Filtered Solution (MBq)**	**Filter/(Filter + Filtered Solution) (%)**
3 µm	91.9	1.13	98.8
5 µm	92.0	1.31	98.6
8 µm	86.0	1.46	98.3
10 µm	88.6	1.49	98.3

## Data Availability

Not applicable.
